# Supplementary material for: Impacts of Mutations in the P-Loop on Conformational Alterations of KRAS Investigated with Gaussian Accelerated Molecular Dynamics Simulations
Source: Molecules. 2023 Mar 23;28(7):2886. doi: 10.3390/molecules28072886 (PMC10095679; doi:10.3390/molecules28072886)
Supplement: Supplementary file 1 [file molecules-28-02886-s001.zip › molecules-2261646-supplementary.pdf]

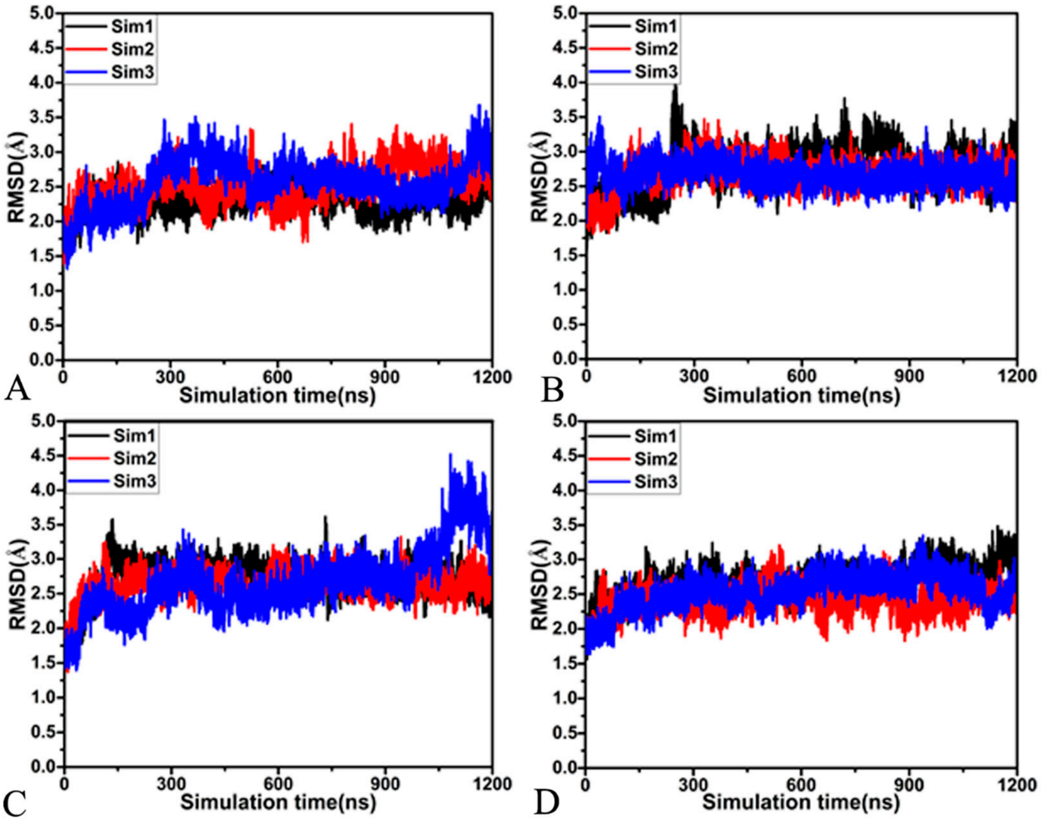

Figure S1. Time evolutions of RMSDs for non-hydrogen atoms of the GDP-bound WT, G12A, G12D and G12R KRAS in three independent GaMD simulations: (A) the GDP-bound WT KRAS, (B) the GDP-bound G12A KRAS, (C) the GDP-bound G12D KRAS and (D) the GDP-bound G12R KRAS.

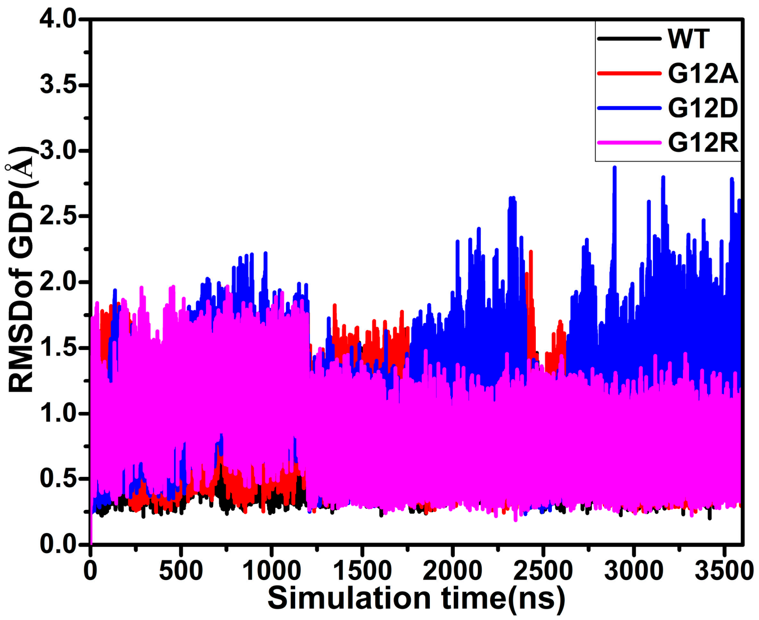

Figure S2. Time evolutions of RMSDs for non-hydrogen atoms of GDP through the entire GaMD simulations.

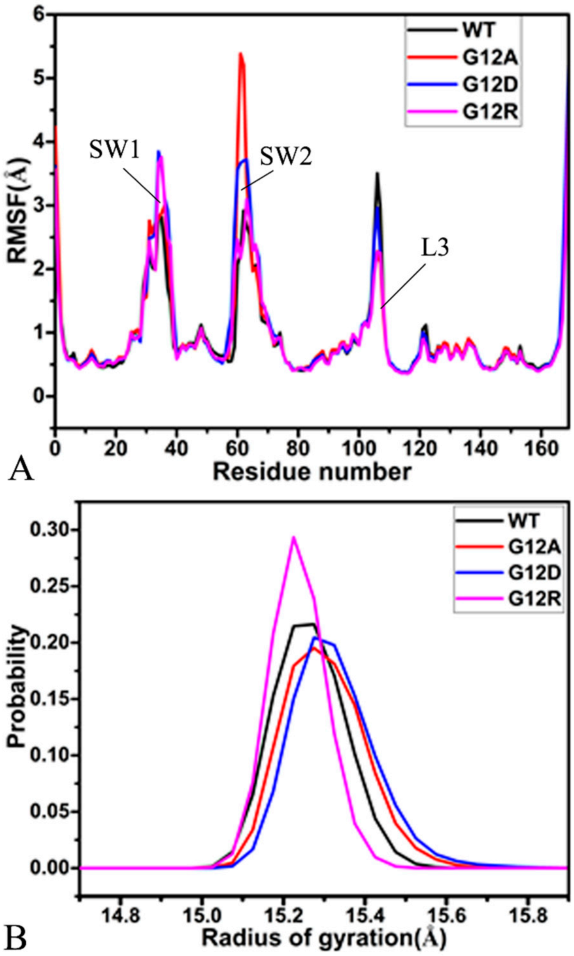

Figure S3: (A) RMSFs of the GDP-bound WT, G12A, G12D and G12R KRAS calculated by using the coordinates of the C_α atoms and (B) probability distribution for radius of gyrations.

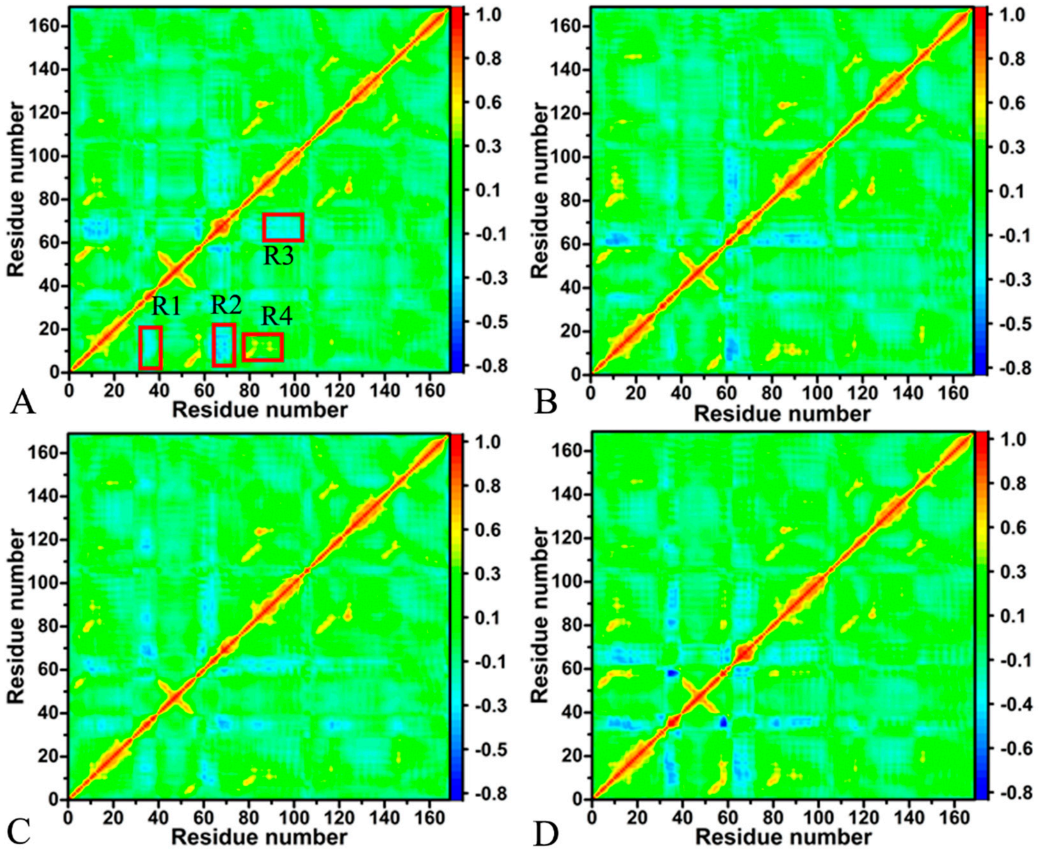

Figure S4. DCCMs of the GDP-bound WT and mutated KRAS computed by using the coordinates of the C_α atoms: (A) the GDP-bound WT KRAS, (B) the GDP-bound G12A KRAS, (C) the GDP-bound G12D KRAS and (D) the GDP-bound G12R KRAS.

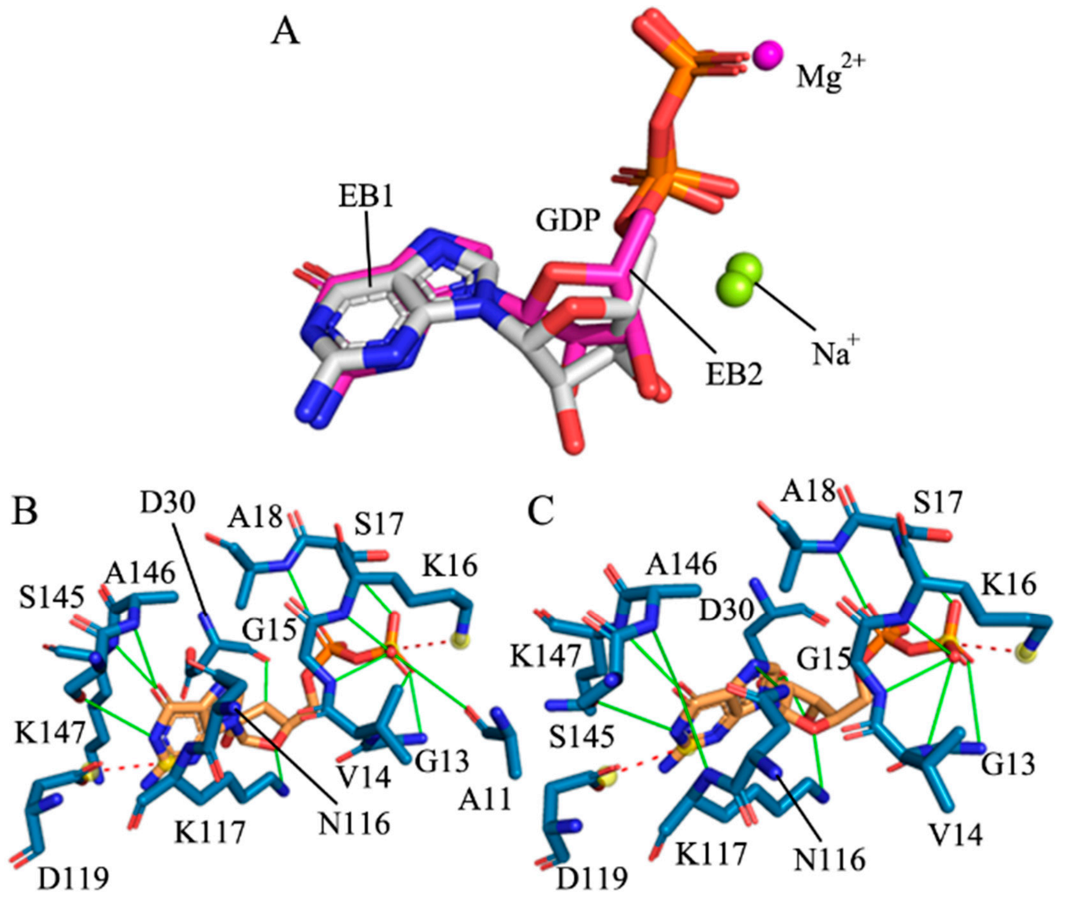

Figure S5. Structural information of the GDP-bound WT KRAS: (A) structural superimposition of GDP and magnesium ions in the structures EB1 and EB2, (B) interactions of GDP with KRAS in the structure EB1 and (C) interactions of GDP with KRAS in the structure EB2.

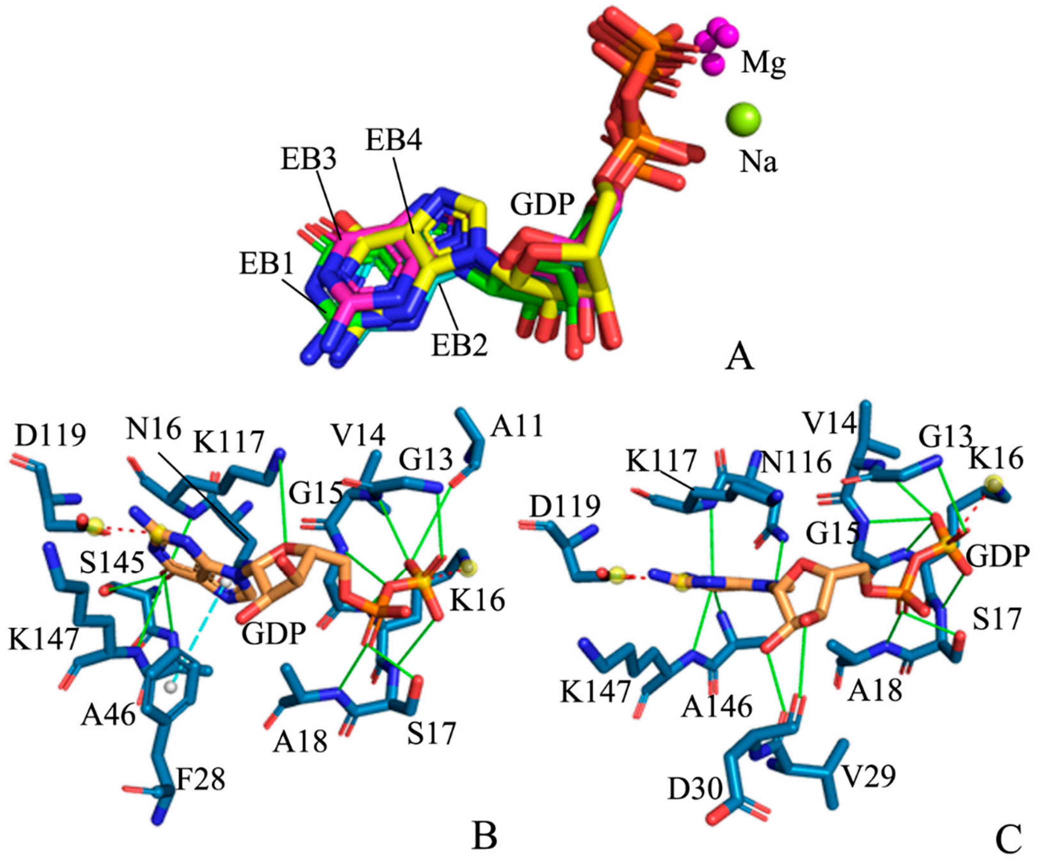

Figure S6. Structural information of the GDP-bound G12A KRAS: (A) structural superimposition of GDP and magnesium ions in the structures EB1-EB4, (B) the interaction of GDP with KRAS in the structure EB1 at the most incompact state of the switch domains and (C) the interaction of GDP with KRAS in the structure EB4 in the tightest state of the switch domains.

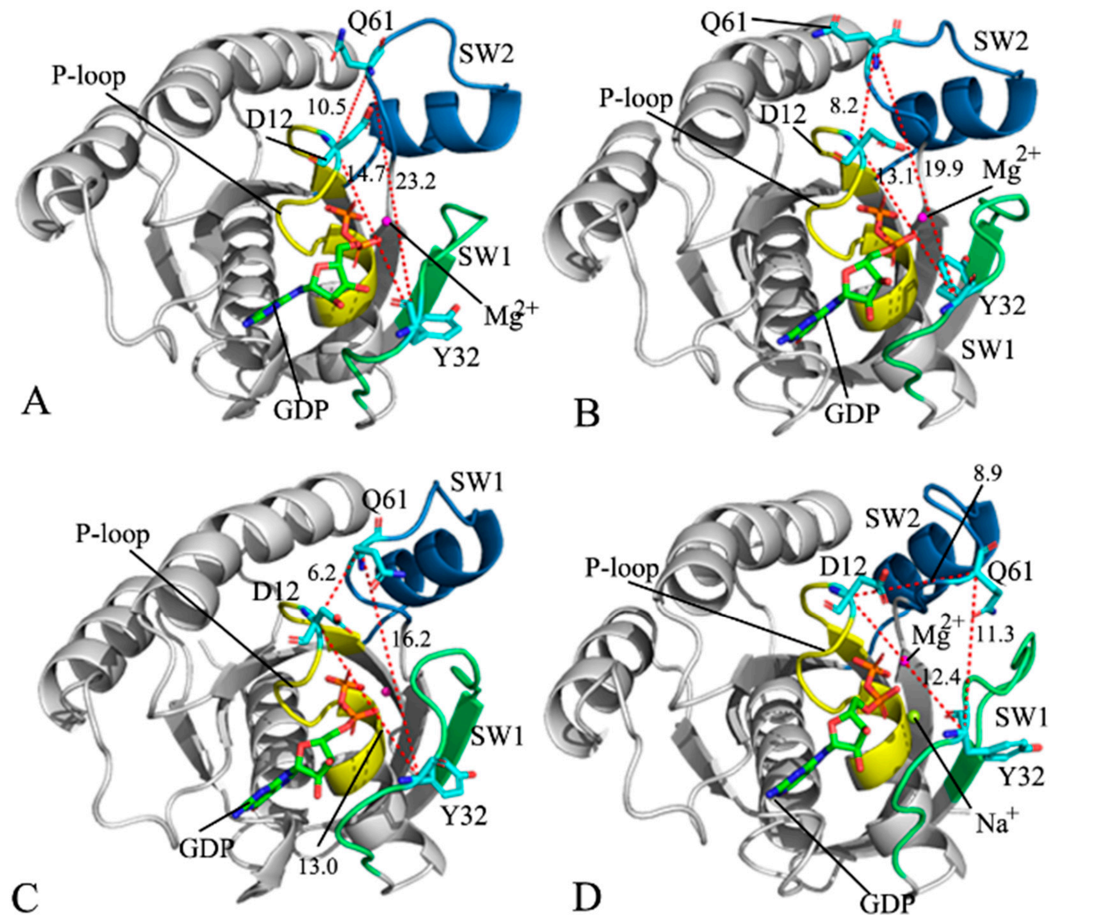

Figure S7. Representative structures of the GDP-bound G12D KRAS situated at the EB1-EB4: (A) the EB1, (B) the EB2, (C) the EB3 and (D) the EB4.

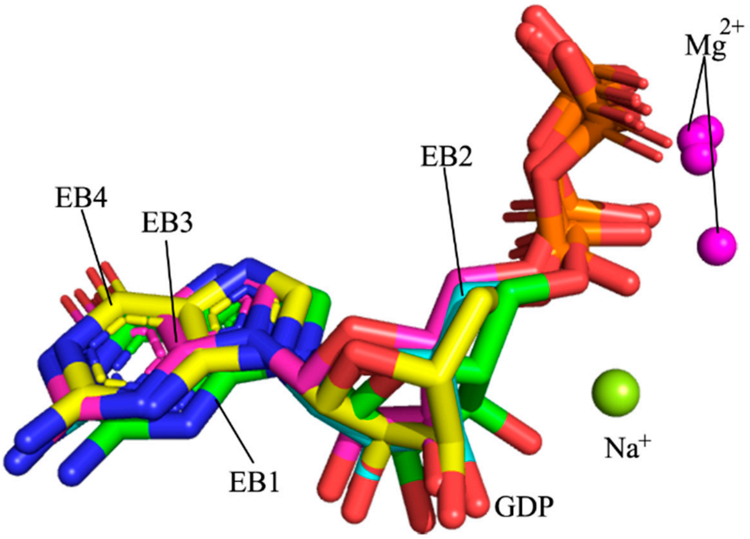

Figure S8. Structural superimposition of GDP and magnesium ions located at the EB1-EB4.

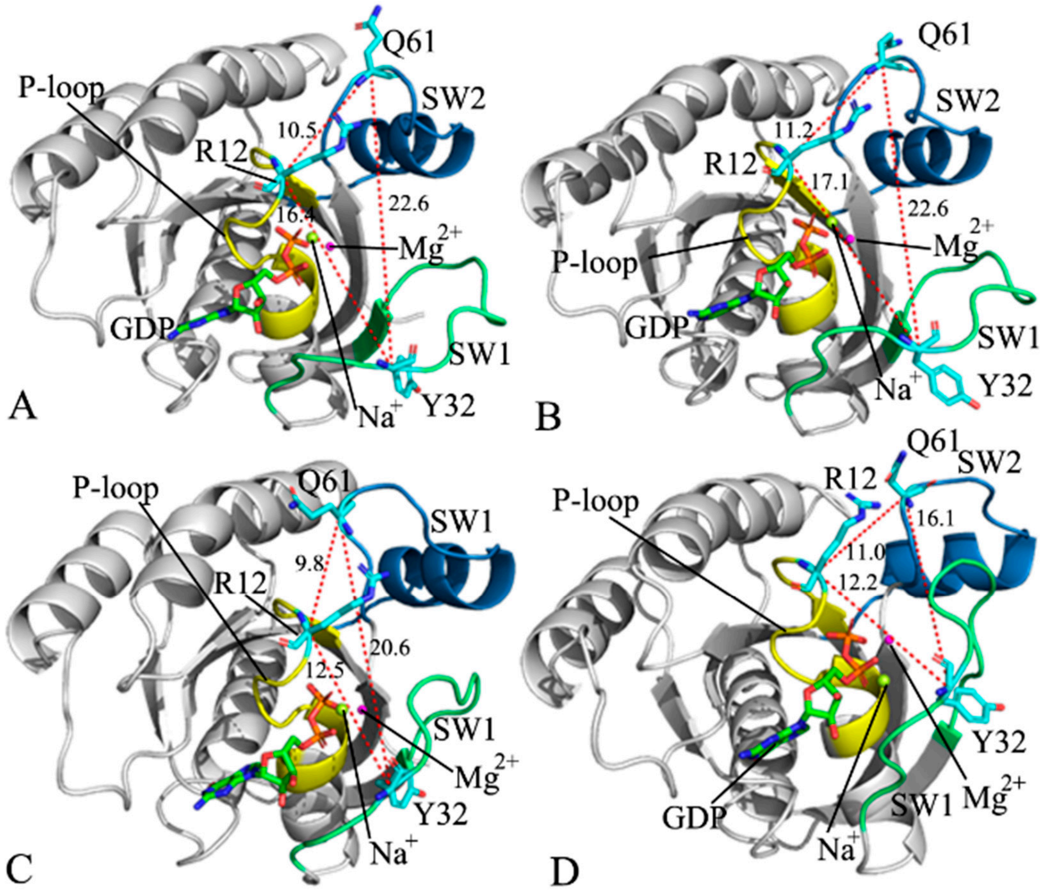

Figure S9. Representative structures of the GDP-bound G12R KRAS situated at the EB1-EB4: (A) the EB1, (B) the EB2, (C) the EB3 and (D) the EB4.

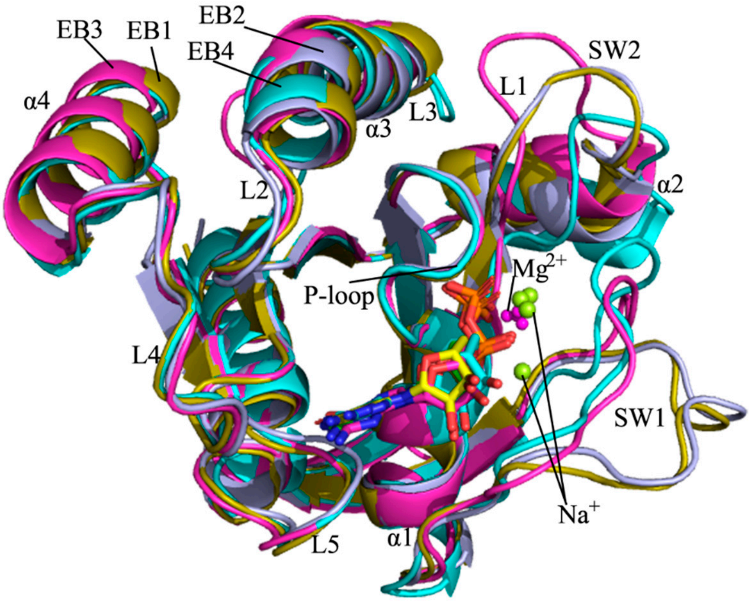

Figure S10. Structural superimpositions of the GDP-bound G12R KRAS located at the EB1-EB4.

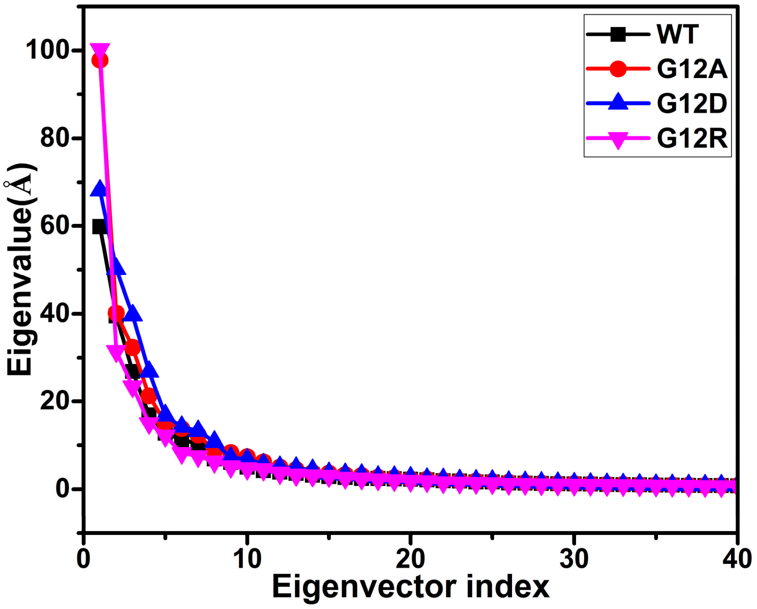

Figure S11. The function of the eigenvalues as the eigenvector index arising from principal component analysis.
